# Proteochemometric modeling of HIV protease susceptibility

**DOI:** 10.1186/1471-2105-9-181

**Published:** 2008-04-10

**Authors:** Maris Lapins, Martin Eklund, Ola Spjuth, Peteris Prusis, Jarl ES Wikberg

**Affiliations:** 1 Department of Pharmaceutical Pharmacology, Uppsala University, SE-751 24, Sweden

## Abstract

**Background:**

A major obstacle in treatment of HIV is the ability of the virus to mutate rapidly into drug-resistant variants. A method for predicting the susceptibility of mutated HIV strains to antiviral agents would provide substantial clinical benefit as well as facilitate the development of new candidate drugs. Therefore, we used proteochemometrics to model the susceptibility of HIV to protease inhibitors in current use, utilizing descriptions of the physico-chemical properties of mutated HIV proteases and 3D structural property descriptions for the protease inhibitors. The descriptions were correlated to the susceptibility data of 828 unique HIV protease variants for seven protease inhibitors in current use; the data set comprised 4792 protease-inhibitor combinations.

**Results:**

The model provided excellent predictability (*R*^2 ^= 0.92, *Q*^2 ^= 0.87) and identified general and specific features of drug resistance. The model's predictive ability was verified by external prediction in which the susceptibilities to each one of the seven inhibitors were omitted from the data set, one inhibitor at a time, and the data for the six remaining compounds were used to create new models. This analysis showed that the over all predictive ability for the omitted inhibitors was *Q*^2 ^_*inhibitors *_= 0.72.

**Conclusion:**

Our results show that a proteochemometric approach can provide generalized susceptibility predictions for new inhibitors. Our proteochemometric model can directly analyze inhibitor-protease interactions and facilitate treatment selection based on viral genotype. The model is available for public use, and is located at HIV Drug Research Centre.

## Background

Despite huge efforts to prevent the spread of HIV, its prevalence continues to increase. Currently over 40 million persons are infected with HIV, and more than 4 million become infected and almost 3 million die from AIDS every year [1]. Intensive treatment with antiretroviral drug combinations has substantially prolonged patient survival. However, the virus is prone to rapid mutation and drug resistant strains emerge, particularly in patients in whom the replication of the virus is only partially suppressed by treatment. The high rate of HIV mutation presents a challenging clinical problem, even a non-treated patient can host many viral variants from which drug resistant strains may evolve once therapy is instituted.

A major pharmacological target in HIV is its protease. The HIV protease is a dimeric protein composed of two identical 99-amino-acid monomers. The protease cleaves the viral Gag-Pol polyprotein, which is a necessary step in the generation of new virus particles. Thus, the HIV protease is essential for the propagation of the virus; nine of the 28 anti-HIV drugs and combination regimens in current use target the HIV protease. However, soon after the introduction of the HIV protease inhibitors it was found that the virus accumulates mutations in the protease, permitting eventual escape from anti-viral therapy. As protease inhibitors differ in their resistance profiles a proper selection of the inhibitor can aid therapy in such cases of drug resistance. The PhenoSense susceptibility test is a widely used bioassay for measuring viral survival during specific drug treatment [2,3], and this assay is used to develop a proper treatment strategy for individual patients.

A more straightforward and cost-effective method for formulating a therapeutic strategy would be to predict drug susceptibility directly from the HIV genome sequence. Several types of modeling approaches have been developed, variously based on neural networks [4], support vector machines [5,6], and other methods [6-8]. A drawback with all of these approaches was that they treated each anti-retroviral drug separately; each inhibitor required a separate model. Accordingly, none of these models can predict the effectiveness of a new drug for mutated proteases. However, such predictions are possible using our proteochemometric approach [9,10]. Proteochemometrics utilizes the physico-chemical and structural properties of series of ligands and proteins to predict their interaction [10]. Proteochemometrics has been successfully used to model various classes of G-protein coupled receptors [9,11-17], antibodies [18], as well as aspartate proteases' ability to cleave their substrates [19]. Here, we show that proteochemometrics can be used to model HIV protease resistance.

## Results

### Development of a proteochemometric model for drug susceptibility prediction

We described seven protease inhibitors using six orthogonal descriptors derived from rotation- and superimposition-independent 3D structure descriptors (I block) while the proteases were described by 240 z-scale descriptors representing physico-chemical properties of 80 varied sequence positions in the data-set (P block; see *Methods *for details). We created several models from these descriptions in order to find the one that provided the highest predictive ability and interpretability. *Model-1 *used protease and inhibitor descriptors (P+I blocks, comprising 240 + 6 = 246 **X **variables); *Model-2 *used protease and inhibitor descriptors and protease-inhibitor cross-terms (P+I and P × I blocks, totaling 246 + 6 × 240 = 1,686 **X **variables); *Model-3 *used an additional 28,680 intra-protease cross-terms (i.e. P+P, P × I, and P × P blocks, totaling 1,686 + 28,680 = 30,366 **X **variables). Models were created from these data by state-of-the-art proteochemometric partial least-squares projections to latent structures (PLS) modeling using the log fold-decrease in susceptibility ("log*FDS*") compared to a drug-sensitive reference virus as estimated using the PhenoSense assay as the **Y **variable (see *Methods *for details); Table 1 summarizes the performances of these models.

**Table 1:** Performance of proteochemometric models for HIV-1 protease drug susceptibility predictions.

Model	Descriptor blocks	Goodness of fit (*R*^2^)	Predictive ability (*Q*^2^)	RMSEP*	Results of permutation test	
					*R*^2 ^intercept	*Q*^2 ^intercept
*Model-1*	P+I	0.75	0.72	0.44	0.02	-0.08
*Model-2*	P+I, P × I	0.86	0.82	0.35	0.14	-0.19
*Model-3*	P+I, P × I, P × P	0.91	0.87	0.30	0.21	-0.27

* Root mean Squared Errors of Prediction

While all models were statistically valid, *Model-2*, which included protease-inhibitor cross-terms, performed substantially better than *Model-1*, which contained only protease and inhibitor descriptors. Adding intra-protease cross-terms (*Model-3*) provided further improvement. Results from permutation testing also indicated the statistical validity of the models. Thus, for none of the models did the *Q*^2 ^intercept show a positive value, ensuring that the high original *Q*^2 ^values were not obtained by pure chance.

As seen in Table 1, adding new descriptor blocks resulted in more positive values for the *R*^2 ^intercepts (although they remain below the desired level of 0.3), confirming that an increase in the number of **X **variables often results in better-fitted models in which part of the **y **data becomes explained by accumulated chance-correlations. Still, the models' predictive ability and interpretability improves because *Q*^2 ^values increase (in contrast to its intercept for randomized data) and root mean squared errors of prediction (RMSEP) values decrease (Table 1). Thus, according to this analysis, *Model-3 *is the best performer. The good performance of *Model-3 *was further demonstrated by its true outer cross-validation; its external predictive ability amounted to *P*^2 ^= 0.85.

### External validation of the drug susceptibility model

In order to validate our approach further, we assessed whether our model could predict susceptibility to inhibitors excluded from the model building. We used *Model-3 *to create seven different models, but only used data for six inhibitors (excluding one inhibitor at a time from the data set), and used each model to predict the susceptibilities to the respective excluded inhibitor for the 828 mutated protease variants (see *Methods *for details). Table 2 shows the RMSEP computed from all predictions in this analysis; the RMSEP of susceptibility predictions is below 0.5 logarithmic units for all inhibitors. Computing the predictive ability for "new" inhibitors from all the seven models from this analysis gave the very high estimate for *Q*^2 ^_*inhibitors *_= 0.72.

**Table 2:** External predictions by proteochemometric HIV-1 protease susceptibility models.

Inhibitor	RMSEP*
Amprenavir	0.48
Atazanavir	0.45
Indinavir	0.33
Lopinavir	0.39
Nelfinavir	0.49
Ritonavir	0.38
Saquinavir	0.49

* Root mean Squared Errors of Prediction

**Figure 1:**
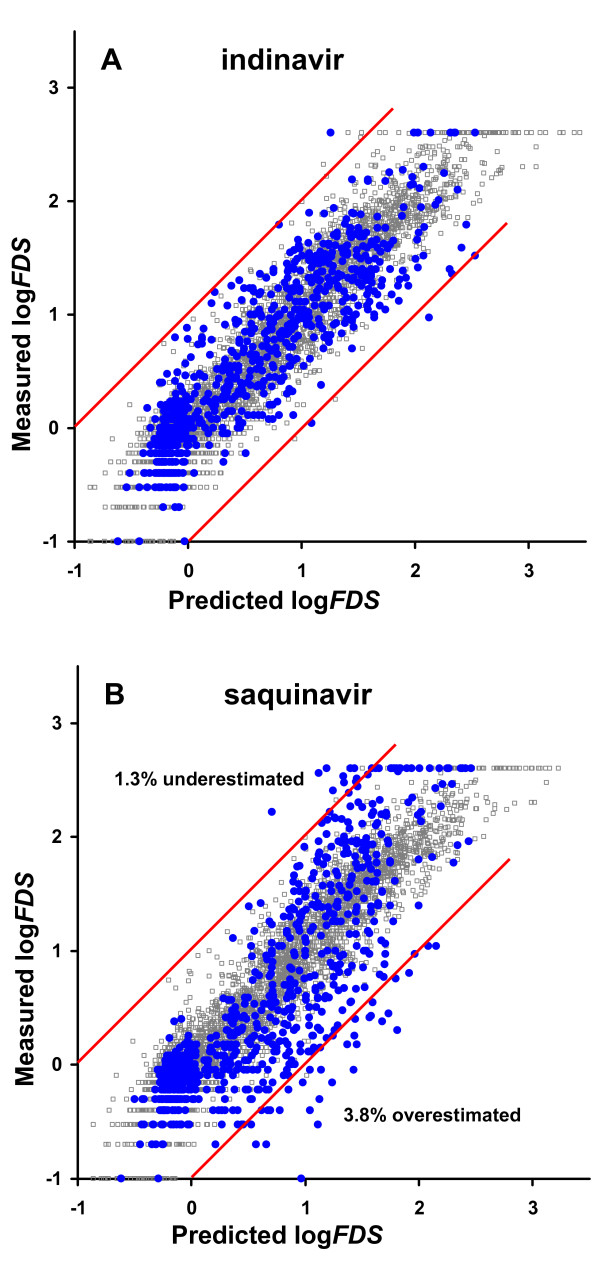
Graphical illustration of the external predictive ability of proteochemometric models for HIV-1 protease drug susceptibility. Data for one inhibitor at a time were excluded from the dataset and predicted from proteochemometric models built on the remaining data. The predicted versus measured susceptibility values for indinavir (A) and saquinavir (B) are shown. Goodness-of-fit of the models (i.e. model data) are shown as light gray symbols in panels A and B.

Figure 1 shows the predicted versus measured susceptibility for the inhibitor with the smallest prediction error, indinavir (RMSEP = 0.33; Panel A) and the inhibitor with the largest prediction error, saquinavir (RMSEP = 0.49; Panel B). The predictions for indinavir correlate excellently with the measured susceptibilities. For saquinavir, the susceptibilities are underestimated ten-fold for less than 4% percent of all virus isolates, while only 1.3% percent are overestimated more than ten-fold.

These data substantiate the validity of *Model-3 *and its modeling approach; therefore, we used *Model-3 *for all subsequent predictions and interpretations.

### Use of the drug susceptibility model to analyze the role of individual amino acids in drug resistance

The PLS algorithm we used for model building derives a linear regression equation in which the coefficients reveal the direction and magnitude of the influence of **X**-variables on the response variable (i.e. protease inhibitor susceptibility). A large absolute value of a coefficient for a protease descriptor (i.e. a coefficient for a z-scale encoding the physico-chemical properties of amino acids at a particular position; see *Methods *for details) indicates that mutations at this position could induce a large change in drug susceptibility. Alternately, a large absolute value for a coefficient of a cross-term of protease-protease inhibitor descriptor reveals that mutations of the amino acid included in the cross-term can induce large changes in the susceptibilities for some particular inhibitors and not-so-large changes in the susceptibilities for other inhibitors. Finally, a large absolute value of a coefficient for an intra-protease cross-term pinpoints mutations in the protease that regulate drug resistance in a cooperative manner (for a deeper discussion and mathematical derivations relating to this discussion, see [20]).

**Figure 2:**
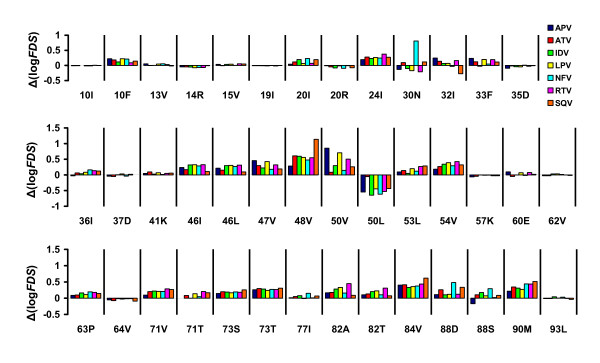
Changes in the susceptibility to the seven inhibitors due to single point mutations in the wild-type HIV-1 protease. Shown are the decimal logarithms of the fold-decreases in susceptibility (FDS) calculated from the proteochemometric model.

However, there are problems associated with the use of regression coefficients for model interpretation because several descriptors represent each sequence residue (i.e., the three *z*-scales used herein) and each of these is used to compute six protease-ligand cross terms, as well as a very large number of intra-protease cross-terms. Therefore, looking at the regression coefficient for one variable at a time does not provide full insight about the influence of a particular residue to the susceptibility of the protease to a protease inhibitor. One simple approach to this complex situation is to use the whole regression equation to predict the susceptibility of *in silico *mutated variants of the protease. We used this approach to predict changes in susceptibilities due to single point mutations in the wild-type protease (HIV-1 subtype B consensus reference sequence; Figure 2). The figure presents the 35 most frequent mutations occurring in over 5% of viral isolates in the data set, as well as six somewhat less common mutations that we found to be important by the proteochemometric modeling (namely 32I, 47V, 50V, 50L, 53L, and 73T). As seen from the figure, many common mutations do not negatively influence susceptibility to any of the inhibitors. Many of these mutations are polymorphic, that is, they occur in untreated patients and thus reflect natural variation in the protease, such as 10I and 20R [21]. In contrast, the 10F and 20I mutations are non-polymorphic; they occur only in response to drug treatment. As shown in Figure 2, these two mutations reduce protease susceptibility to several inhibitors, and several other mutations impart high resistance to particular drugs. For example, the 30N mutation causes a large decrease in susceptibility to nelfinavir, while it has no effect on susceptibility to the other inhibitors. Another example is the 48V mutation, which substantially decreases susceptibility to saquinavir while having only a minor effect on susceptibility to amprenavir. On the other hand, the 50V mutation confers major resistance to amprenavir, and to a lesser extent, to lopinavir and ritonavir, while having essentially no influence on susceptibility to the remaining four inhibitors. A large number of other mutations have distinct effects on different inhibitors; in some cases they even increased susceptibility to some inhibitors (for example, 30N increases the susceptibility to ritonavir and 32I to saquinavir). Another interesting example is the 50L mutation, which occurs in response to treatment with atazanavir and increased susceptibility to the six other inhibitors used in the model.

Several different mutations (24I, 54V, 73T, 84V, and 90M) reduce susceptibility to all seven inhibitors, although there is variation in the extent of the reduction for specific inhibitors. Moreover, our analysis revealed that the more recently introduced inhibitors lopinavir and atazanavir do not provide increased activity as compared to older protease inhibitors against protease variants bearing these mutations. These findings indicate the need for novel, more adaptive agents that can inhibit proteases harboring these deleterious mutations.

### Online prediction of susceptibility resulting from accumulated mutations

Highly resistant forms of HIV protease evolve by accumulating multiple susceptibility-decreasing mutations. The good predictive abilities of *Models-1 *and *2 *indicate that the logarithmically transformed susceptibility data is, to a large extent, a function of the additive independent contributions of each mutation. However, *Model-3*, which also included intra-protease cross-terms, showed even better predictive ability suggesting that mutations may also interact cooperatively to modify drug resistance.

Complete analysis of the contributions of all possible combinations of amino acid mutations and protease inhibitors to drug resistance is an extensive task and could not be presented easily in a written account such as this. Therefore, we made our model available to the public in form of a Web service, so that users can submit their protease sequence and receive a prediction of drug susceptibility. The use of a Web service makes facilitates integration in other applications and workflows. Access to the Web service is available at HIV Drug Research Centre [22].

**Figure 3:**
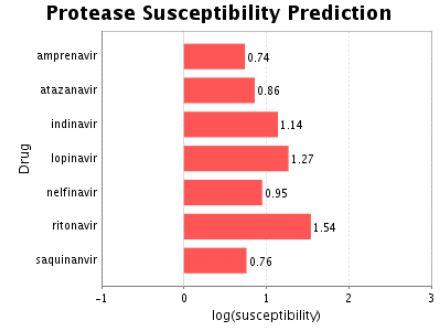
Screenshot from the Web service for the proteochemometric susceptibility model of HIV protease inhibitors. The publicly available prediction service takes an HIV protease sequence as input and predicts its susceptibility to seven protease inhibitors using the proteochemometric model. The output is graphical and indicates any anomalies in the submitted sequence with respect to the data in the model. Shown are results for a protease with the quadruple mutation 24I, 46L, 54V, and 82A. The Web service can be found at [22].

Figure 3 shows a screenshot of output from the Web service illustrating the predicted susceptibilities for a protease containing mutations 24I, 46L, 54V, and 82A Separately, these mutation convey moderate (up to three-fold) reductions in susceptibility to any of the seven protease inhibitors (Figure 2). However, when combined these mutations cause a sizable increase in resistance to several of the inhibitors, the predicted decrease in susceptibility to ritonavir is almost 40-fold. For saquinavir and amprenavir, there is only about five- to six-fold decrease in susceptibility (Figure 3).

## Discussion

We used proteochemometrics to model susceptibilities of multiple HIV-1 protease variants to seven clinically used protease inhibitors, yielding a model with very good predictability and interpretability. We thoroughly validated the external predictive ability and the statistical significance of the model estimates, and conclude that our model can be reliably applied to the prediction and interpretation of the mechanisms of drug resistance. In fact, our model shows much better goodness-of-fit and predictability (in terms of *R*^2^, *Q*^2 ^and RMSEP) than the hitherto best-performing models reported elsewhere, which, to the best of our knowledge, were obtained by the use of Support Vector Machines applied to each protease inhibitor separately [6].

Our model uses physico-chemical property (z-scale) descriptors of amino acids rather than encoding the mutations by letter codes or binary indicator variables. This is highly advantageous as many sequence residue positions of the HIV protease are often mutated to amino acids that share similar physico-chemical properties. Our model can evaluate the contribution of each encoded property (e.g. hydrophobicity, steric properties, charge, etc.) to drug susceptibility and perform predictions for mutations to any amino acid, as long as the amino acid's properties fall within the scope of the model. In other words, the information gained from sequence positions with multiple mutations provides for predictions for novel mutations at the same position.

**Figure 4:**
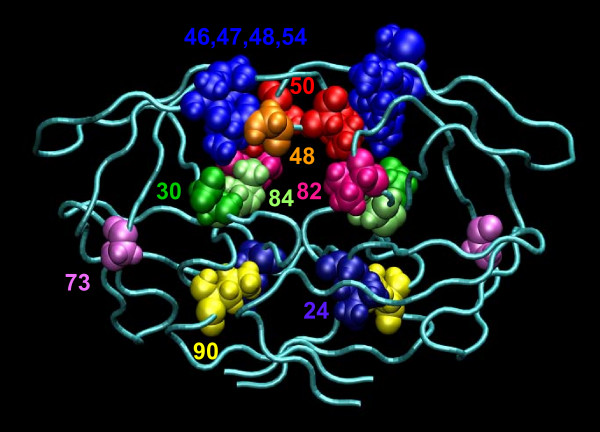
Locations of 12 major drug susceptibility-reducing mutations in the HIV-1 protease identified by the proteochemometric model based on the analysis in Figure 2.

The model allowed us to identify the mutations that contribute most to resistance to current protease inhibitors (see Figure 2). Most of these mutations (such as 47V, 48V, 82A, and 84V) are located in the active site of the protease (Figure 4). Other deleterious mutations, such as 90M, are located outside the binding site. The 90M mutation is located in the dimerization region of the HIV protease. Historically, such mutations have been regarded as "compensatory", compensating for the negative effects of active-site mutations on the conformation and dimerization of the protease, and its ability to cleave substrates. However, more-recent studies suggest that distantly located mutations might diminish the ability of inhibitors to bind the protease by indirectly altering the geometry of the active site [23,24]. Proteochemometrics makes a powerful impact on our ability to detect, analyze, and predict the contributions of such indirect interactions to ligand binding [20]. Effects arising from distant residues are often overlooked in drug design because they are difficult to account for using 3D docking or other methods.

Besides identifying mutations that contribute to general resistance to protease inhibitors, the model also reveals the susceptibilities of particular combinations of protease inhibitors and protease mutants. Thus, the model identifies specific relationships between a particular inhibitor and a particular amino acid(s) in the protease, information that could be useful for analyzing the mechanisms behind inhibitor failure. Moreover, the predictive ability of the model enables the development of targeted treatment based on the genome of a particular viral variant.

It is here appropriate to mention that an alternative way of resistance to protease inhibitors is mutations of the Gag-Pol cleavage sites which lead to enhancement of the processing efficiency of the substrate [25]. Unfortunately the Stanford HIV Drug resistance database does not provide the full genomes of the HIV isolates, and this precludes us to linking the present data-set to complementary mutations in the cleavage sites. However, it seems unlikely that cleavage site mutations play any major role in explaining drug susceptibility in the present case. This is because our model, which was based on protease and inhibitor chemical properties alone, explained over 90% of the variation in the data, where the unexplained variation presumably essentially just represents measurement errors.

In this study we encoded the 3D structures of protease inhibitors by so called GRIND descriptors. These descriptors provide quantitative characterization of the ability of a molecule to form H-bond donor/acceptor and hydrophobic interactions with pharmacophoric groups located at various distance ranges around the molecule. Moreover, we also used a recently-developed GRIND descriptor type (TIP) which describes differences in size and shape of the molecules. These descriptions thus account for all major types of interaction that may contribute to inhibitor binding within the HIV protease, as well as those that can destroy binding (e.g. by steric hindrances). PCA was then applied to compress the descriptions into six orthogonal principal components, and in this case (obtaining six components for seven inhibitors) the PCA did not discard any of the information in the original descriptors. Accordingly the PCAs used herein allow a complete back-tracing to their origin in the original descriptors [13]. An advantage of using GRINDs is that they do not require alignments of molecules and thus are not limited to narrow series of congeneric compounds. On the other hand, difficulties may arise in model interpretations since structural modifications in the molecule often influence the values of multiple GRIND descriptors. A practical approach in the design of improved compounds in such a situation is to predict changes of susceptibility patterns for *in-silico *modified molecules. For example, such predictions suggest that the loss of susceptibility of nelfinavir to the D30N mutant should be possible to counteract by modifying the 3-hydroxy-2-methylphenyl group of the compound (data not shown). In other words, according to these predictions the resistance to nelfinavir arises due to less favorable interactions of the 3-hydroxy-2-methylphenyl group with the mutated protease compared to its interactions with the wild type protease. Thus our modeling approach may find use to predict susceptibilities to new inhibitors and could potentially be applied in design of new inhibitors. Current susceptibility data is limited to the few clinically used protease inhibitors but proteochemometric modeling could be applied in a more general fashion and aid in the design of new agents with improved ability to withstand the development of resistance.

## Conclusion

In summary, proteochemometrics is well suited to the study of HIV protease drug resistance. Our model predicts that relatively few of the more common mutations contribute substantially to a general loss of susceptibility, suggesting that there are limits as to how the virus can escape from the inhibitors. Whether the capacity of the protease to mutate into drug resistant variants is restricted due to inherent biological factors or whether new mutations would appear in response to broader chemical diversity of protease inhibitors remains to be determined. The appearance of new mutations in response to new treatments would require repeated agglomerative modeling of susceptibility data. Analysis of larger datasets (comprising more chemical compounds and more viral variants) would improve the resolution and predictive ability of the proteochemometric model and consequently augment its potential application to drug design and therapy optimization.

## Methods

### Data set

Susceptibility data for the seven clinically used HIV-1 protease inhibitors amprenavir, atazanavir, indinavir, lopinavir, nelfinavir, ritonavir, and saquinavir, measured by the PhenoSense assay [2], were collected from the Stanford HIV Drug Resistance Database [26]. In short, the PhenoSense assay estimates the concentration of the anti-HIV drug that causes 50% inhibition of an HIV isolate's replication in a cell-based assay. The fold-decrease in susceptibility (here abbreviated as *FDS*) was determined by dividing this concentration by the concentration of the drug causing 50% inhibition of a drug-sensitive reference virus (the wild-type strain NL4-3). Thus, *FDS *= 1 indicates unchanged susceptibility to a drug, while *FDS *> 1 indicates decreased susceptibility, that is, increased resistance of the tested isolate as compared to the standard.

We retrieved susceptibility values for 4,794 unique protease-inhibitor pairs (comprising 828 unique protease sequences) from the database. For five of the seven inhibitors, susceptibility data was available for 775 to 824 proteases. For atazanavir and lopinavir, which are more recently approved protease inhibitors, susceptibility data was available for 319 and 513 proteases, respectively.

### Numerical descriptions for proteochemometric modeling

#### Description of proteases

Of the 99 amino acid positions in each protease monomer, 80 were found to be mutated in the data set. Mutated positions were encoded by three *z*-scale descriptors, z_1_-z_3_, of amino acids derived by Sandberg *et al. *[27]. The three *z*-scales are based on 26 computed and measured physico-chemical properties of amino acids, and represent hydrophobicity, steric properties, and electronic properties of amino acids, yielding 80 × 3 = 240 protease descriptors.

#### Description of protease inhibitors

3D structures of organic compounds were generated using Corina software (Molecular Networks GmbH, ), and were described by grid independent descriptors (GRINDs) [28] calculated using Almond 3.1 software (Multivariate Infometric Analysis S.r.l., ). GRINDs are alignment-independent descriptors that relate to the ability of a molecule to form favorable interactions with independent pharmacophoric groups. Three groups were used: DRY (hydrophobic), O (H-bond acceptor), and N1 (H-bond donor). The overall shape of the molecule was represented by a "TIP-field" using a recently described approach in which the region with repulsion energy of 1 kcal/mol for the N1 group is used to outline the surface of the molecule [29].

Generation of GRIND descriptors involves several steps: (1) calculation of interaction energies of the molecule with pharmacophoric groups located at grid points surrounding the molecule; (2) calculation of distances between grid points; (3) grid filtering (this is performed by selecting a certain number of grid nodes showing most favorable interactions with the molecule and concomitantly being situated as far as possible from each other); and, (4) computing the products of energy values for all pairs of the selected grid nodes. Finally, the maxima of products falling within specified distance ranges for node pairs obtained using the same probe (DRY, O, N1, and TIP auto-correlograms) and different probes (DRY-O, DRY-N1, DRY-TIP, O-N1, O-TIP, and N1-TIP cross-correlograms) are used as descriptors for the molecules [28]. Thus, the capabilities of the protease inhibitors for hydrophobic, H-bond donor, and H-bond acceptor interactions and the differences in the molecular shapes of the protease inhibitors were encoded by 545 GRIND descriptors. To reduce the number of descriptors and to eliminate their mutual co-linearity, we applied principal component analysis [30], which transformed all GRINDs into six orthogonal principal components.

#### Protease-inhibitor and intra-protease cross-terms

Protein-ligand interactions are governed by complex processes that depend on the complementarity of the properties of the interacting entities. In proteochemometrics, this is accounted for by computing protein-ligand cross-terms [13]. In order to account for effects of particular mutations on the susceptibility to particular inhibitors, we computed cross-terms by multiplying mean-centered z-scale descriptors with mean-centered principal components of GRINDs. This yielded 1,440 (240 × 6) protease-inhibitor cross-terms. To account for eventual co-operative coupling of mutations in the protease [31], we introduced intra-protease cross-terms. These were computed by multiplying mean-centered *z*-scale descriptors, which gave 240 × 239/2 = 28,680 cross-terms.

#### Preprocessing of data

Since descriptors were of different origins, they were centered and scaled to unit variance prior to their use. Moreover, to account for differences in the number of descriptors and their formed cross-terms, block scaling was applied. The block scaling was applied onto three descriptor blocks, namely, the block formed from ordinary protease and inhibitor descriptors (P+I block), the block composed of protease-inhibitor cross-terms (P × I block), and the block composed of intra-protease cross-terms (P × P block). Block scaling was performed by systematically varying the standard deviation of P+I block descriptors in one standard deviation intervals and the standard deviation of P × P block descriptors in 0.3 standard deviation intervals until an optimal model was obtained.

The dependent variable (*FDS*) was logarithmically transformed and mean centered prior to use in the computations.

### Proteochemometric modeling

#### Correlation by partial least-squares projections to latent structures

The above-derived descriptors and cross-terms were correlated to the susceptibility data by using the partial least-squares projections to latent structures (PLS). In PLS, the independent matrix of **X **variables (i.e., all descriptors and cross-terms) and a matrix of one or several dependent variables **Y **(in our case the logarithms of the *FDS *values comprise a single **y **vector) are simultaneously projected to latent variables (PLS components), with an additional constraint to maximize the covariance between the projections of **X **and **Y **(for an in-depth review of the PLS see [32]). PLS derives a regression equation for each response **y **in which the regression coefficients reveal the direction and magnitude of the influence of **X**-variables on the response.

#### Validation of modeling

The goodness-of-fit of the PLS models was characterized by the fraction of explained variation of the **Y **(*R*^2^). The predictive ability was characterized by the fraction of the predicted **Y**-variation (*Q*^2^), assessed by cross-validation with seven randomly formed groups, as previously described [33]. The *R*^2 ^values vary between 0 and 1, where a higher value means a better fit. The *Q*^2 ^values normally vary between 0 and *R*^2^; however, negative values may be encountered, indicating non-predictive models. In PLS, the *R*^2 ^term increases with each extracted PLS component, while the *Q*^2 ^value usually reaches a plateau and declines as the model becomes over-fitted. Hence, the predictive ability and not the goodness-of-fit should be used when assessing the optimal number of PLS components.

In addition to the conventional "inner" cross-validation, we performed outer cross-validation in which the entire modeling process (description, scaling, and PLS fit) was performed independently from the excluded data; that is, it corresponded to the modeling practice of the "training set" and "test set" of the data but was performed seven times on random selections of data [34]. The performance of outer cross-validation was assessed by the *P*^2 ^value, which is calculated in the same way as *Q*^2 ^of the inner cross-validation.

To assess the statistical significance of the estimated *Q*^2 ^and *R*^2 ^values, we employed permutation testing [35,36]. The susceptibility data was randomly reordered 20 times, and separate models were fitted, correlating **X **data to each of the permuted **y**. The results of permutation testing can be displayed by plotting the *R*^2 ^and *Q*^2 ^values of these models of partially random data versus the correlation coefficient between the original **y **and permuted **y**, and drawing the regression line [36]. The intercepts of the regression lines (that is, when the correlation coefficient is zero) represent the *R*^2 ^and *Q*^2 ^of a purely random model. To affirm full statistical significance of the original estimates the desirable limits of intercepts should be *R*^2 ^intercept <0.3 and *Q*^2 ^intercept <0.05 [36].

We also wanted to assess the ability of the proteochemometric model to predict susceptibility to novel inhibitors that were not present in the model in any combinations with the mutated proteases. Therefore, we removed all data for one inhibitor at a time and fitted the model for the remaining six inhibitors. **X **variables were re-centered and rescaled to unit variance and the **y **variable was re-centered prior to PLS modeling. The predictions for the excluded inhibitors were calculated from PLS models created on the reduced datasets and assessed by the RMSEP estimate.

## Authors' contributions

ML performed the majority of the modeling, data analysis, and model interpretations. PP contributed to early modeling. ME and OS coded the prediction server. JESW initiated and supervised the project. All authors contributed to drafting the manuscript.
